# Breeding a novel cauliflower with exceptional fragrance

**DOI:** 10.1186/s43897-025-00178-8

**Published:** 2025-07-02

**Authors:** Xiaoli Zhang, Xiaoxu Li, Long Chen, Zhenghua Wen, Fengqing Han, Daping Gong, Minmin Xie, Zhe Zhao, Yu Zhao, Wei Zhang, Mingli Chen, Zhiyuan Li

**Affiliations:** 1 Tobacco Research Institute, Chinese Academy of Agricultural Sciences, Qingdao, China; 2 State Key Laboratory of Vegetable Biobreeding, Tianjin Academy of Agricultural Sciences, Tianjin, China; 3 Beijing Life Science Academy, Beijing, China; 4 State Key Laboratory of Vegetable Biobreeding, Institute of Vegetables and Flowers, Chinese Academy of Agricultural Sciences, Beijing, China; 5 Technology Center of China Tobacco Hunan Industrial Co. Ltd., CNTC, Changsha, China; 6 Technology Center, China Tobacco Shandong Industrial Co., Ltd, Jinan, China

Cauliflower (*Brassica oleracea* var. *botrytis* L.), a cruciferous vegetable belonging to the *Brassicaceae* family, is a vital component of global vegetable production systems. Renowned for its high nutritional value, which includes substantial levels of protein, lipids, carbohydrates, dietary fiber, vitamins, and minerals, it provides significant health benefits. Taste components, particularly the aroma, influence the culinary and consumption experience, thereby affecting human food preferences (Lu and Zhu 2022). Current breeding initiatives prioritize the development of novel cultivars with enhanced organoleptic traits while maintaining yield stability, addressing the growing demand for premium horticultural commodities in global fresh produce markets.

2-Acetyl-1-pyrroline (2-AP) is a notable aromatic compound that imparts a distinctive “popcorn-like” fragrance to various crops. Research has demonstrated that the biosynthesis of this compound is regulated by the enzyme betaine aldehyde dehydrogenase (BADH). Genetic polymorphisms in the *BADH* gene have been successfully utilized in breeding programs for multiple crops, including rice (Chen et al. 2008; Okpala et al. 2019), maize (Wang et al. 2021), sorghum (Yundaeng et al. 2013; Xie et al. 2024), foxtail millet (Zhang et al. 2022), soybean (Qian et al. 2022) and tobacco (Chen et al. 2024a). Previous research has indicated that the fragrant aroma of natural aromatic rice can be attributed to variations in the *OsBADH2* gene rather than the *OsBADH1* gene (He et al. 2015). Notably, the experimental outcomes observed in corn, sorghum, millet, and tobacco strikingly parallel those documented in rice, suggesting a conserved mechanism across these divergent plant species. However, the role of *BADH* genes in 2-AP accumulation in soybean differs slightly from that in the aforementioned crops. In soybean, *GmBADH2* predominantly regulates the 2-AP content, while *GmBADH1* also significantly contributes to this regulation. Furthermore, simultaneous knockout of both *GmBADH* genes can lead to an increase in 2-AP content (Xie et al. 2024).

Previous studies on the regulation of 2-AP content by *BADH* genes in crops have focused predominantly on seeds, with limited research focused on floral organs. Notably, the *BADH* genes associated with the 2-AP biosynthetic pathway remain uncharacterized in cauliflower, a vegetable crop distinguished by its edible inflorescence. Consequently, functional characterization of *BADH* genes within this pathway is essential for facilitating quality-driven breeding strategies for cauliflower improvement.

In our study, two *BADH* genes (*BOB06G101460* and *BOB03G089620*) were identified in the cauliflower genome (Chen et al. 2024b) through comprehensive genomic analysis. A phylogenetic tree of BADH proteins from cauliflower and other plants was constructed via the neighbor‒joining method, which revealed clear evolutionary divergence between the *BoBADH* genes and their homologs (Table S1). One of the *BADH* genes (*BOB06G101460*) was most closely related to *ALADH10A8* (*AT1G74920*) and was named *BoBADH1*, whereas the other *BADH* gene (*BOB03G089620*) was closely associated with *ALADH10A9* (*AT3G48170*) and was consequently named *BoBADH2* (Figure S1). We then designed specific primers to amplify the full-length *BADH* coding sequences (Table S2). The *BoBADH1* and *BoBADH2* genes were found to contain open reading frames (ORFs) with lengths of 1,509 bp and 1,512 bp, respectively. Both of these *BADH* genes are predicted to contain 15 exons. Protein sequence alignment revealed significant homology (79% identity) between BoBADH1 and BoBADH2, suggesting functional redundancy while distinct evolutionary trajectories were retained. To investigate biological functions, CRISPR/Cas9-mediated knockout was performed using hypocotyl explant transformation in the cauliflower inbred line QN-52(WT). Two sgRNAs targeting exon 2 of the *BoBADH* gene were cloned and inserted into the CRISPR/Cas9 binary vector pBSbdcas9i and introduced into QN52 via *Agrobacterium*-mediated transformation. Through PCR amplification, primary screening identified 41 positive transgenic plants in the T0 generation. On the basis of the sequencing analysis, 36 genes were found to have mutations in either or both target genes. We then selected homozygous T1 generation lines for further experiments. Among these lines, *bobadh1-3* had a 23-bp deletion in *BoBADH1,* and *bobadh1-4* had a 1-bp deletion in *BoBADH1*, whereas *bobadh2-2* had a 10-bp deletion in *BoBADH2,* and *bobadh2-7* harbored a 7-bp deletion in *BoBADH2*. Among the *bobadh1/2* double-mutant lines, *bobadh1/2–4* harbored a 1-bp deletion in *BoBADH1* and a 7-bp deletion in *BoBADH2*, whereas *bobadh1/2–9* contained a 1-bp deletion in *BoBADH1* and a 10-bp deletion in *BoBADH2*. Notably, all of these mutations were frameshift mutations in *BoBADHs* (Fig. 1A-C).

**Fig. 1:**
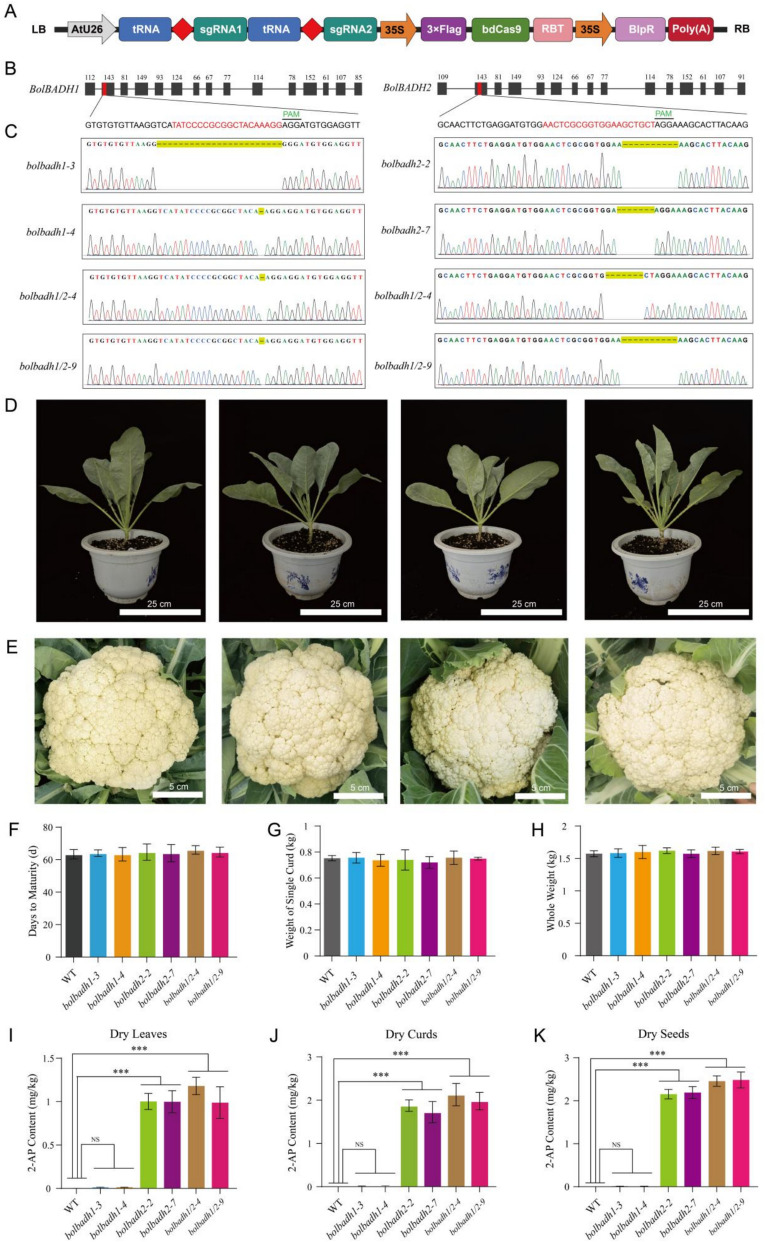
CRISPR/Cas9‐mediated editing of *BoBADH1* and *BoBADH2* in cauliflower. **A** Schematic of the CRISPR/Cas9 construct. **B** Gene structure of *BoBADH1* and *BoBADH2* and the design of sgRNAs for *BoBADH1* and *BoBADH2*. The black rectangles represent exons, the black lines represent introns, and the red rectangles represent target sites. **C** Mutation analysis of edited *BoBADH1* and *BoBADH2*. **D** Photograph of QN-52 (WT), *bobadh1*, *bobadh2* and the *bobadh1/2* double mutant at 56 days after transplanting. **E** Curd of QN-52 (WT), *bobadh1*, *bobadh2* and the *bobadh1/2* double mutant. **F**-**H** Maturity time (**F**), weight of a single curd (**G**), and whole weight (**H**). **I**-**K** 2-AP contents of dry leaves (**I**), dry curds (**J**) and dry seeds (**K**). The data in (**F**-**K**) are shown as the means ± SEs (*n*=3). *P* values were calculated via *t* tests (**F**‒**H**) or ANOVA (**I**‒**K**). ***P < 0.001

The growth parameters of the WT and transgenic plants, including maturity time, total biomass, and single-curd weight, were systematically assessed. Statistical analyses revealed no significant differences between single/double-mutant lines and the WT. These findings demonstrate that targeted editing of *BoBADH* genes does not disrupt normal growth or developmental processes in cauliflower, thereby providing critical evidence for implementing CRISPR-based strategies for flavor trait enhancement without compromising yield (Fig. 1D-H).

To validate the fragrance phenotype of the *BADH*-edited lines, we performed a sensory evaluation coupled with 2-AP quantification. The fresh leaves were sheared and then immersed in a 1.7% (w/v) KOH solution for 15 minutes (Chen et al. 2024a). Organoleptic assessment revealed a distinct popcorn-like aroma in *BoBADH2* single mutants and *BoBADH1/2* double mutants, whereas neither *BoBADH1* single mutants nor WT plants emitted a detectable fragrance. Quantitative analysis of 2-AP content across tissues (leaves, curds, and seeds) was conducted via gas chromatography‒mass spectrometry (GC‒MS) (Shan et al. 2015). As expected, we detected 2-AP only in *BoBADH2* single mutants and *BoBADH1/2* double mutants, with the 2-AP content ranging from 0.855 to 2.683 mg/kg. However, only trace amounts of 2-AP were detected in the *BoBADH1* single mutants and the WT, ranging from 0.001 to 0.025 mg/kg. These findings indicate that only the *BoBADH2* gene, not the *BoBADH1* gene, is responsible for conferring fragrance in cauliflower (Fig. 1I-K).

Furthermore, resequencing data from 820 *Brassica oleracea* germplasm accessions were downloaded from the NCBI SRA database under BioProject accession number PRJNA794342 (Chen et al. 2024b). Systematic screening of the *BoBADH2* gene was then conducted to identify loss-of-function mutants. Following the exclusion of materials with suboptimal sequencing quality, frameshift mutations in the *BoBADH2* gene were detected in two accessions (PN_0816 and PN_0818), both of which presented homozygous genotypes. A popcorn-like aroma was detected in these accessions via organoleptic assessment, which indicated that frameshift mutations result in loss of function of the *BoBADH2* gene. Overall, these loss-of-function mutants provide invaluable germplasm resources for the molecular breeding of cauliflower cultivars with enhanced aroma.

In conclusion, we successfully identified two *BADH* genes (*BoBADH1* and *BoBADH2*) in the cauliflower genome. Single/double-mutant lines of *BADH* genes were generated via the CRISPR/Cas9 system. Our study revealed that *BoBADH2*, but not *BoBADH1,* is essential for 2-AP accumulation in the leaves, seeds and curds of cauliflower. Fragrance can be an attractive feature to consumers of cauliflower, an important vegetable with a high nutritional content, thus increasing its market value, as observed for fragrant rice. Overall, our research lays the groundwork for developing aroma-enriched cauliflower varieties through molecular breeding technologies.

## Supplementary Information

Supplementary Material 1: Supplementary Figure S1. Phylogenetic tree depicting the evolutionary relationships among members of the BADH protein family in plants

Supplementary Material 2: Supplementary Table S1. Information on the proteins used for phylogenetic tree construction

Supplementary Material 3: Supplementary Table S2. Primers used in this study

## Data Availability

The datasets and materials that support the findings of this study can be obtained by contacting the corresponding author. The original data in this study are included in the paper and its supplementary materials.
