# Supplementary material for: Breeding a novel cauliflower with exceptional fragrance
Source: Mol Hortic. 2025 Jul 2;5:56. doi: 10.1186/s43897-025-00178-8 (PMC12220755; doi:10.1186/s43897-025-00178-8)
Supplement: Supplementary file 1 — Supplementary Material 1: Supplementary Figure S1. Phylogenetic tree depicting the evolutionary relationships among members of the BADH protein family in plants [file 43897_2025_178_MOESM1_ESM.pdf]

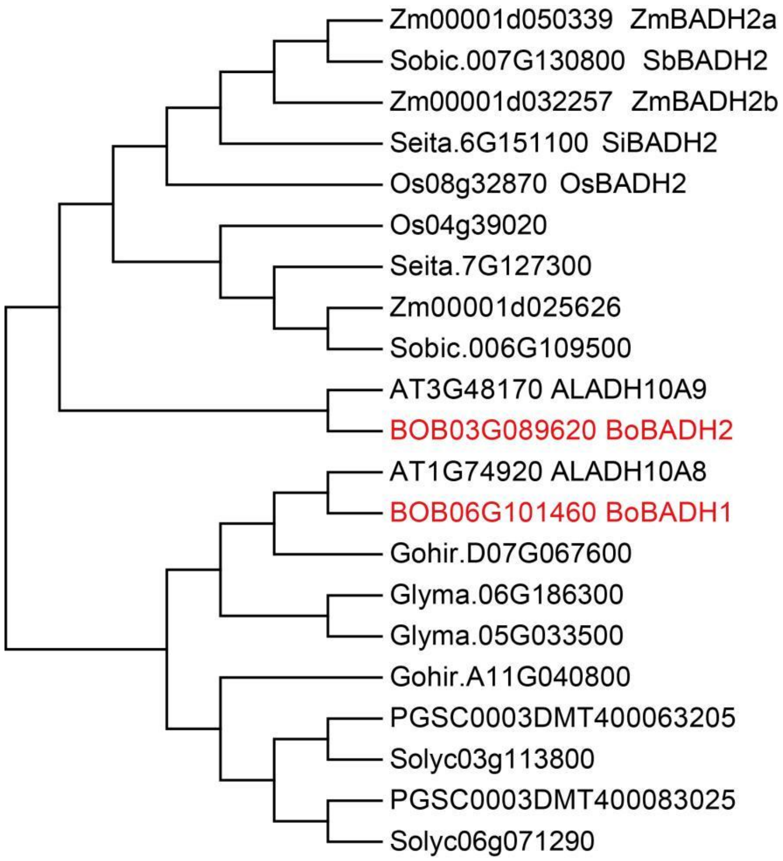

Figure S1. Phylogenetic tree depicting the evolutionary relationships among the BADH protein family in plants.
